# Cystic echinococcosis amongst small ruminants and humans in central Ethiopia

**DOI:** 10.4102/ojvr.v82i1.949

**Published:** 2015-08-21

**Authors:** Habtamu Assefa, Belay Mulate, Shahid Nazir, Alula Alemayehu

**Affiliations:** 1 School of Veterinary Medicine, Wollo University, Ethiopia

## Abstract

This study was conducted to determine the prevalence of cystic echinococcosis (CE) in small ruminants and humans in Addis Ababa, central Ethiopia. A cross-sectional study involving systematic random sampling was conducted to estimate the prevalence of CE in 512 small ruminants (262 sheep and 250 goats) slaughtered at Addis Ababa Abattoir Enterprise between October 2011 and March 2012. Hydatid cysts were identified macroscopically during postmortem examination and their fertility and viability were determined. CE was observed in 21 (8.02%) sheep and 17 (6.80%) goats. In sheep 13 (4.96%) of the lungs, 10 (3.81%) livers and 1 (0.381%) heart were found to be infected with hydatid cysts. Involvement of lung and liver in goats was found to be 10 (4.0%) and 8 (3.2%) respectively, with no cysts recorded in the heart. Of the total of 77 and 47 cysts encountered in sheep and goats, 33 (42.85%) and 15 (31.91%) respectively were fertile. Viability of protoscoleces from fertile cysts in sheep (29 [87.87%]) was higher than in goats (6 [40.0%]). For humans, retrospective analysis covering five years of case reports at two major hospitals in Addis Ababa between January 2008 and December 2012 showed that of the total of 25 840 patients admitted for ultrasound examination, 27 CE cases were registered, a prevalence of 0.1% and mean annual incidence rate of approximately 0.18 cases per 100 000 population. Liver was the major organ affected in humans (81.5% in affected patients) followed by spleen (11.1%) and kidney (7.4%). Logistic regression analysis showed that prevalence of CE varied significantly in relation to host age in the small ruminants (OR = 3.93, *P* < 0.05) as well as in humans (95% CI, *R* = 4.8). This epidemiological study confirms the importance of CE in small ruminants and humans in central Ethiopia, emphasising the need for integrated approaches to controlling this neglected preventable disease.

## Introduction

Cystic echinococcosis (CE) caused by larval stages of *Echinococcus granulosus* is one of the most common zoonotic diseases associated with severe economic losses and great public health significance worldwide (Romig *et al.* 2011). *Echinococcus* infections are estimated to affect approximately two to three million people worldwide, with Africa amongst the primarily endemic regions (Cummings, Rodriguez-Sosa & Satoskar 2009). Dogs and wild canids (wolves, jackals, red foxes and others) are the primary definitive hosts, with ungulates (sheep, goats and camels) as intermediate hosts and humans are aberrant intermediate hosts (Eckert & Deplazes 2004). Intermediate host species accidentally ingest infective eggs that develop into a metacestode stage in different organs (liver, lungs and kidneys), which is referred to as CE. The cycle is completed when an intermediate host or its infected organ is eaten by a suitable carnivore (Carmena, Sánchez-Serrano & Barbero-Martínez 2008). The adult tapeworm in the definitive host is harmless, unlike the hydatid cyst in the intermediate host animals – that is of immense economic and medical importance in the infected host (Azlaf & Dakkak 2006; Ibrahim 2010).

Humans are infected by ingesting eggs of *E. granulosus* through contaminated food, water and soil, or through direct contact with dogs (Pednekar *et al.* 2009). The role of dogs in the spread of the disease has also been reported by other researchers in Ethiopia (Endrias, Yechale & Assefa 2010; Jobre *et al.* 1996; Jones *et al.* 2011). Since the infection rate in dogs is directly proportional to the fertility of cysts (Urquhart *et al.* 2003), cyst characterisation in the intermediate host is important to assess the infectivity of the parasite. CE in humans has frequently been reported from different regions of the country (Erbeto, Zewde & Kumsa 2010). The disease is more common in rural areas, where dogs and domestic animals live in very close association (Fromsa & Jobre 2011). Most CE cases in humans are caused by the sheep strain (GI) and camel strain (G6) of *E. granulosus* (Japhet, Ernest & Eberhard 2006). In humans the cyst may reside and grow in liver, lung and other visceral organs. Occasional rupture of the cysts often leads to sudden death because of anaphylaxis, haemorrhage and metastasis (Getaw *et al.* 2010; White, Peter & Weller 2004).

CE in livestock causes considerable economic losses due to condemnation of affected animal organs at the slaughterhouse, production losses (reduction in live weight gain, yield of milk, fertility rates, value of hide and skin) and losses related to treatment of animals and humans (Torgerson 2003). The economic burden of CE on the global livestock industry alone has been estimated to be over $2 billion per annum (Scala *et al.* 2006). Such losses are of particular importance in Ethiopia, which has low economic output with a per capita income of less than one US dollar per day.

In Ethiopia, where home slaughter of cattle, sheep, goats and camels is still practised and uncooked offal and carcass wastes are normally fed to dogs and cats, CE has become an endemic disease and poses public health problems. Although a lot of work has been done on CE in bovines in Ethiopia (Endrias *et al.* 2010; Getaw *et al.* 2010; Kebede, Mitiku & Tilahun 2009b; Kebede *et al.* 2011), only a few published reports are available on CE in small ruminants and humans. Moreover, small ruminants play an important role in the life cycle of *E. granulosus* due to higher fertility and viability of hydatid cysts in these animals (Kumsa & Mohammedzein 2012). This study was therefore undertaken to investigate the prevalence of and risk factors for CE in small ruminants slaughtered at Addis Ababa Abattoir Enterprise, and to estimate the occurrence of CE in humans in Addis Ababa, Ethiopia, based on hospital records.

## Research method and design

### Setting

The study was conducted between October 2011 and March 2012 at Addis Ababa Abattoir Enterprise, and also included a retrospective study of CE at two human hospitals (hospital 1 and hospital 2) in Addis Ababa, capital city of Ethiopia. Addis Ababa is situated at 11°1’48”N and 39°37’59.83”E, at an altitude of 2000 m a.s.l. – 3000 m a.s.l. The city has a subtropical highland climate. The mean annual rainfall is 1800 mm with a bimodal pattern, whilst the daytime mean annual minimum and maximum temperatures are 14 °C and 21 °C respectively (Central Statistical Agency 2010). The short rainy season occurs from February to March, and the long rainy season starts from the end of June and ends during early November. The farming system in the area is mixed livestock and crops, and sheep are the dominant animal species reared by farmers. The total area of the city is about 527 km^2^ and in 2010 the total human population was estimated to be 2 917 295 (Central Statistical Agency 2010).

### Cystic echinococcosis in small ruminants

#### Study population

The study was undertaken in both sexes and all age groups of local breed sheep and goats slaughtered for human consumption at Addis Ababa Abattoir Enterprise, which was established 60 years ago in the heart of Addis Ababa and is the biggest abattoir in the country. The study animals were brought from different parts of the country for slaughter (Borena, Wellita, Hararghe, Gamogofa, Kambeta, Hadiya, Gurage, Debre Birhan, Awash, Matahara, Afar, Wollo, Tigray, Shewarobit, and Addis Ababa and surrounds). On average 700 cattle, 250 sheep and 75 goats were slaughtered daily during the study period.

#### Study design and sample size

A cross-sectional study was conducted on 512 small ruminants (262 sheep and 250 goats) slaughtered at Addis Ababa Abattoir Enterprise to determine the prevalence of CE from postmortem examination of visceral organs, which included liver, lungs, heart, kidneys, spleen, mesentery, peritoneum and omentum. A systematic random sampling procedure was employed to carry out this study. The sample size for each animal species required for this study was determined based on the expected prevalence (19.94% for sheep and 16% for goats) of CE (Erbeto *et al.* 2010) and the 5% desired absolute precision and 95% confidence interval (CI) according to Thrusfield (2005):

$$n=\frac{1.96^{2}Pexp\left(1-Pexp\right)}{d^{2}}$$ [Eqn 1]

Where *n* = sample size, Pexp = expected prevalence (19.94% for sheep and 16% for goats), D = desired absolute precision (5%), and 1.96^2^ = *z*-value at 95% confidence level.

Accordingly, the required sample size was 472 (245 sheep and 207 goats), but in order to increase precision it was maximised to 512 (262 sheep and 250 goats).

Data relating to origin, sex, age and body condition of each study animal were recorded during the survey. The age of each animal was estimated based on the dentition formula as given by Abegaz and Awgichew (2009); sheep and goats with only milk teeth were classed as young (less than 1 year old), and those with one to four pairs of permanent incisors as adult, whereas animals with a full set of teeth were classed as old. The body condition scoring was classified into three categories: lean (1, 2), medium (3), and fat (4, 5), according to Thompson and Meyer (1994).

#### Postmortem examination of small ruminants

In the abattoir thorough meat inspection was carried out on various organs of each of the slaughtered animals, particularly lung, liver, kidney, heart and spleen. Each organ was assessed macroscopically by visual inspection and palpation. One or more incisions were made to detect small hydatid cysts (Soulsby 1982). An organ or carcass was considered infected if it contained one or more hydatid cyst or cysts. The infected organs from each positive animal were collected and the number, size, localisation and type of cyst were recorded.

#### Hydatid cyst characterisation

The individual cysts from each of the infected organs were carefully incised and examined for protoscoleces. The protoscoleces appeared as white dots on the germinal epithelium, and such cysts were characterised as fertile.

The fertile cysts were further subjected to a viability test. This was done by placing a drop of fluid taken from the cyst containing protoscoleces on a glass microscope slide, covering with a cover slip and examining it for amoeboid-like peristaltic movements using a 40X objective. For clear vision a drop of 0.1% aqueous eosin solution was added to an equal volume of protoscoleces in hydatid fluid on the microscope slide, according to the principle that viable protoscoleces should completely or partially exclude the dye whilst the dead ones take it up (Macpherson *et al.* 1985; Smyth & Barrett 1980). Viability of protoscoleces was determined for each fertile cyst per animal species and organ.

Sterile hydatid cysts were identified by their smooth inner lining, usually with a slight turbidity of the fluid content, and by typical calcified cysts that produced a gritty texture upon incision (Parija 2004; Soulsby 1982). Small and completely calcified cysts of less than 5 mm in diameter were not included in this study, since it was difficult to differentiate these from other metacestode lesions.

### Cystic echinococcosis in humans

A five-year retrospective hospital data survey was used to determine the prevalence of CE in humans. The authors reviewed the retrospective data from ultrasound examinations carried out for different purposes during the period January 2008 to December 2012 at hospital 1 and hospital 2 in Addis Ababa. These hospitals had properly maintained patient records and the hospital management was highly cooperative in sharing these, which was the basis for selection of these hospitals for this study. Moreover, these hospitals are considered to be the major referral hospitals in Addis Ababa and provide medical care for a wide area as a reference consultation centre for patients from several primary healthcare centres. Data on human hydatid cysts were identified and the mean annual incidence was determined. Only those cases referred from areas under Addis Ababa’s authority were included in the study.

### Data analysis

Data collected from antemortem, postmortem and laboratory findings were entered into MS Excel; the Statistical Software Package for Social Sciences^®^ (SPSS) version II 5 (SPSS Inc., Chicago, IL, United States of America) was employed to analyse the data. Mean and percentage were used to calculate prevalence. Multivariable logistic regression analysis was conducted to determine the association between risk factors and occurrence of CE in small ruminants. Similarly, annual incidence of human CE (per 105 population) was determined by calculating the number of new registered CE cases in these hospitals per year divided by the average population of Addis Ababa during the five years (2008–2012: 2 917 295). Also, percentage prevalence of CE was determined in humans and odds ratios (OR) were used to measure the differences by age group, sex and between the hospitals. In all of the analyses the confidence level (CI) was 95% and the significance level was 5%.

## Results

Of the total 512 carcasses of 262 sheep and 250 goats examined at Addis Ababa Abattoir Enterprise, 38 (7.42%) were infected with *E. granulosus* metacestodes. Cysts were detected in 21 (8.02%) and 17 (6.80%) of the inspected sheep and goats respectively. The difference in the prevalence of the disease in the two species was not significant (OR = 1.54,* P* = 0.06). Results showed that infection prevalence in small ruminants differed significantly by age (OR = 3.93, *P* = 0.02) and with altitude (OR = 3.5, *P* = 0.04) (Table 1).

**TABLE 1:** Multiple logistic regression analysis of cystic echinococcosis occurrence in small ruminants by species, origin, sex, age and body condition.

Risk factors	Number examined	Number positive	%	Odds ratio	95% CI	*P*-value
**Species**						
Caprine	250	17	6.80	1	-	0.062
Ovine	262	21	8.00	1.54	1.12–1.31	-
**Origin**						
Lowland	243	12	4.90	1	-	0.04
Highland	269	26	9.70	3.5	2.67–4.31	-
**Sex**						
Female	313	22	7.00	1	-	0.73
Male	199	16	8.00	1.8	1.1–22	-
**Age**						
Young	76	7	9.20	2.1	1.23–3.1	0.02
Adult	341	19	5.60	1	-	-
Old	95	12	12.60	3.93	2.58–4.7	-
**Body condition**						
Lean	223	17	7.60	2.3	-	0.98
Medium	216	16	7.40	1.9	1.21–3.3	-
Fat	73	5	6.80	1	-	-
**Total**	**512**	**38**	**7.42**	**-**	**-**	**-**

Examination of organs indicated that livers and lungs were the most frequently infected visceral organs in both species (Table 2). However, there was no significant difference in the prevalence of CE in the two organs in both species (*P* > 0.05). The prevalence of infection in lungs was 4.96% and 4.0% in sheep and goats respectively. Concurrent infection of both the liver and lungs was less common than infections of either liver or lung alone. Only one sheep was detected to harbour a cyst in a different organ, the heart (Table 2). Concurrent infection of both liver and lungs constituted 14.28% (3/21) of cases in sheep and 23.53% (4/17) in goats. On average sheep liver and lung were found to harbour 4.3 and 2.5 cysts per organ respectively, whereas in goats 3.37 and 2 cysts per organ were found in liver and lungs respectively. The mean intensity of occurrence of cysts in the liver was found to be higher than in the lungs of all the examined hosts. The number of cysts in infected animals ranged from 1 to 18.

**TABLE 2:** Fertility and/or sterility of cysts collected from organs of sheep and goats slaughtered at Addis Ababa Abattoir Enterprise.

Species	Organ	Fertile cysts	Fertile cysts (%)	Sterile cysts	Sterile cyts (%)	Total	*X* values†	*P* values†	*X* values‡	*P* values‡
Sheep	Liver	10	23.26	33	76.74	43	χ^2^ = 22.47	P = 0.00	χ^2^ = 5.13	P = 0.0693
	Lung	22	66.67	11	33.33	33				
	Heart	1	100	-	-	1				
	**Total**	**33**	** 42.85**	**44**	**57.14**	**77**				
Goats	Liver	4	14.81	23	85.18	27	χ^2^ = 16.296	P = 0.00		
	Lung	11	55.00	9	45.00	20				
	Heart	-	-	-	-	-				
	**Total**	**15**	**31.91**	**32**	**68.09**	**47**				

†, For fertile cysts between different organs in a particular species; ‡, for fertile cysts between the two species.

There was no significant difference in proportion of fertile cysts in sheep and goats (*χ*^2^ = 5.13, *P* = 0.069). The hydatid cysts from lungs exhibited a significantly higher fertility rate (66.67% in sheep and 55.0% in goats) than those obtained from livers (23.26% in sheep and 14.81% in goats) (Table 2). The percentage of calcified cysts was higher in goats (48.94%; 23/47) than in sheep (25.97%; 20/77). The viability of protoscoleces from fertile cysts of sheep (87.87%; 29/33) was greater than that found in goats (40.00%; 6/15). Similarly, a significant difference was observed between livers and lungs in the number of viable protoscoleces in both species (*P* < 0.05). The viability rate of protoscoleces of the liver in sheep and goats was 70.70% (8/10) and 25% (1/4) respectively, whereas in lung it was 90.90% (20/22) and 45.45% (5/11) respectively.

Of a total of 25 840 patients admitted for ultrasound examination in the two hospitals in Addis Ababa, 27 (0.10%) CE cases were registered between January 2008 and December 2012, giving a mean annual incidence rate of 0.1851 per 100 000 population. Of these, 13 (48.1%) were from hospital 1 and 14 (51.9%) from hospital 2 (Table 3). Age and sex groups of the human hydatid cases are shown in Table 3. Females were much more likely to be infected than males, and the highest prevalence was observed in patients above 40 years of age (OR = 2.87, CI = 1.71–3.89). There was no significant variation in prevalence of CE in the two hospitals (OR = 1.4, CI = 1.12–3.12) Hydatid cysts were most commonly encountered in the liver (81.5%; 22/27) and less frequently in the spleen (11.1%; 3/27) and kidneys (7.4%; 2/27).

**TABLE 3:** Age and sex distribution of cystic echinococcosis cases in two public hospitals in Addis Ababa, January 2008 – December, 2012.

Risk factors	Total number examined	Infected number	Infected %	Odds ratio	95% CI	*P*-value
**Age (yrs)**						
< 10	984	0	-	-	-	-
10–20	6456	3	0.07	1	-	-
21–30	7814	7	0.09	1.68	1.21–3.23	0.041
31–40	8428	7	0.08	1.38	1.11–2.67	-
> 40	4126	10	0.24	4.8	2.23–7.56	-
**Sex**						
Male	11 840	7	0.06	1	-	-
Female	14 109	20	0.14	2.87	1.71–3.89	0.03
**Hospitals**						
Hospital 1	13 932	13	0.09	1	-	-
Hospital 2	11 908	14	0.12	1.4	1.12–3.12	0.68
Total	25 840	27	0.10	-	-	-

## Ethical considerations

Before any attempt to collect data on human cases was made, the protocol was approved by the ethical committee of the College of Health Science and Medicine, Wollo University. Official permission was also obtained from both of the hospitals. Anonymity was guaranteed for all of the records reviewed.

## Discussion

CE is known to be of importance in the health of both livestock and humans, which is not apparent to farmers. Most studies on prevalence of CE in domestic animals have relied on postmortem examination in abattoirs (Acosta-Jamett *et al.* 2010; Ahmadi & Meshkehkar 2011; Ibrahim 2010; Kebede *et al.* 2011), as this is an economical way of collecting and analysing information on livestock diseases, particularly in subclinical cases. Hydatid cysts usually persist for the lifespan of the animal, so infection status can be determined during postmortem examination (Njoroge *et al.* 2000).

The study conducted at Addis Ababa Abattoir Enterprise indicated that there was no significant difference in the prevalence of CE in sheep (8.02%) and goats (6.80%). In Ethiopia the prevalence of CE in small ruminants has also been reported, viz. 22.2% in sheep in Nekemte (Kumsa 1994), 19.9% in sheep in Addis Abeba (Kebede *et al.* 2011), 16% in goats in Addis Ababa (Erbeto *et al.* 2010), 17.7% in sheep and 6.8% in goats in Harmaya (Yeshiwork 2009), 29.3% in sheep and 6.7% in goats in central Oromia (Getaw *et al.* 2010), 8.05% in sheep and 8.99% in goats in Modjo Modern Export Abattoir (Abiyot, Beyene & Abunna 2011), and (29.5%) in sheep and (24.8%) in goats in Jimma Town (Kumsa & Mohammedzein 2012). The difference in the prevalence of CE in sheep and goats in different parts of Ethiopia indicated above might be due to variations in agro-ecology at different study sites, age, breed of study animals, stocking rates, movements of animals, animal husbandry systems, culture and religion of the society, and number of dogs in different regions of the country (Kebede, Mitiku & Tilahun 2010; Kumsa 1994).

For similar reasons the prevalence of CE in sheep and goats differs in other countries of the world; sheep: goat prevalence was reported to be 11%:6% in north central Chile (Acosta-Jamett *et al.* 2010), 12.61%:6.56% in Al Baha region, Saudi Arabia (Ibrahim 2010), 45.52%:10.0% in southern Iran (Oryan *et al.* 2012), 8.85%:6.21% in Pakistan (Iqbal *et al.* 2012) and 16.42%:2.88% in Tunisia (Lahmar *et al.* 2013). The difference in prevalence rates in the countries could also be associated with factors such as control measures put in place, level of community awareness about the disease, education and economic status of the population and the farming community. In addition to this, the difference in prevalences could also be attributed to variation in strains of *E. granulosus* amongst different countries and geographical locations (Ibrahim 2010).

The prevalence of hydatid cysts in the present study was found to be lower than previous findings (19.94% in sheep and 16% in goats) at the same abattoir (Erbeto *et al.* 2010). This may partly be attributed to greater awareness amongst farmers, antihelminthic treatment of dogs, better control measures adopted, grazing areas for the animals and controlled slaughtering measures to avoid stray dogs coming to eat offal. However, no such improvement in awareness, treatment or practices was apparent in reality. Also, in recent years several programmes have been initiated to control rabies, with large numbers of stray dogs eliminated in the region.

Liver and lung were the most commonly infected organs, and this could be due to the fact that these are the first large capillary fields encountered by the blood-borne oncospheres before any other peripheral organ is involved (Kebede *et al.* 2009a). Location of cysts and cyst morphology is influenced not only by host factors but also by parasite factors, such as the strain of *E. granulosus* involved (Eckert & Deplazes 2004). All of the cysts encountered in both sheep and goats were unilocular, indicating the cystic stage of *E. granulosus*, which is by far the most common found in food animals (Gracey 1986).

In the present survey old animals (with a broken mouth) were found to have significantly higher infection rates than others. This finding is in agreement with the results of Azlaf and Dakkak (2006) and Kebede *et al.* (2009a). This could mainly be due to the fact that old animals will have had longer exposure to the eggs of *E. granulosus,* in addition to weak immunity due to old age (Himonas 1987). Also, the chances of detecting cysts during meat inspection are higher in older animals due to the larger size of the cysts (Baswaid 2007). The significantly high prevalence of CE in small ruminants of highland origin may be due to the low environmental temperatures that favour survival of eggs of the parasite. Changes in the environment and epidemiological factors can also affect rate of transmission of CE (Abiyot *et al.* 2011).

Fertility of the cysts in domestic herbivores provides a reliable indicator of the importance of each type of animal as a potential source of infection to dogs. The fertility rate of cysts in both species in this study was found to be significantly higher in lungs compared to the liver, in agreement with Kebede *et al.* (2009a) and Regassa, Molla and Bekele (2010). It has been reported that the relatively softer consistency of lung tissue allows easier development of the cyst (Himonas 1987). In contrast to the present study, Ibrahim (2010) found higher fertility rates of hepatic cysts than pulmonary ones in small ruminants. Due to the high proportion of fertile cysts (42.85%) and viability of protoscoleces (87.87%), sheep may be considered to be an important intermediate host for *E. granulosus* in Ethiopia. The difference in fertility and the proportion of viable protoscoleces from fertile cysts may be related to the difference in immunological response reflected by the host. Moreover, the fertility of hydatid cysts in the intermediate hosts may also be dependent on the genotype of the parasite (McManus 2006). Unfortunately detailed studies on hydatid cyst genotypes per host in this region are scanty. The liver was shown to harbour a greater number of small calcified cysts, which could be attributed to a relatively higher population of reticulo-endothelial cells and abundant connective tissue reaction in this organ (Kumsa & Mohammedzein 2012).

The endemic nature of CE in small ruminants in Ethiopia could be attributed to a high population of carnivores, particularly stray dogs, in the grazing area of domestic ruminants, and lack of proper efforts to segregate domestic and wild carnivores from livestock or their grazing areas. The habit of feeding ruminant offal to dogs also enhances completion of the life cycle of the parasite. Wild carnivores, especially hyenas, jackals and foxes, may also play a role in the transmission dynamics of *E. granulosus*, particularly in the highland areas where large populations of these animals are found scavenging around human settlements.

Ultrasonography is considered to be the method of choice for detection of both hepatic and extrahepatic echinococcal cysts in humans (Balik *et al.* 2001). Currently information on the prevalence of human CE in Ethiopia is scanty as the disease is not considered a critical medical condition, and is not even a notifiable disease. The overall mean annual incidence rate of 0.18/100 000 reported here may represent an underestimate of the actual incidence for several reasons. Firstly, this incidence does not take into account patients who underwent ultrasonography at hospitals not included in this study. Secondly, not all patients infected with CE undergo ultrasonography, as there are asymptomatic cases of CE. The estimate from this study complements previous estimates on the global burden of CE in other countries of 2.3–8.5 (per 100 000 population) in north–central Chile (Acosta-Jamett *et al.* 2010), 9.3 in Italy (Mastrandrea *et al.* 2012), 3.4–4.6 in Algeria (Shambesh 1997), 2.3 at Bahir Dar, Ethiopia (Kebede *et al.* 2010), and 0.5% – 1.6% in southern Ethiopia (Eckert *et al.* 2002).

A significantly higher incidence of CE was seen in female patients, in agreement with the findings of Blancas *et al.* (2007). A considerable proportion of females in the Ethiopian community is involved in activities related to farming and herding livestock, so more likely to be affected by the disease due to environmental contamination. Egg transmission to humans can also occur via contaminated hands after handling infected definitive hosts (Deplazes & Eckert 2001).

In the present study the highest incidence of CE was observed in the age group of above 40 years, which indicates that the rate of infection of CE increases as the age of the patient advances. Also, people in this age group are probably the most active in livestock rearing. The relatively higher incidence of CE in this age group limits their ability to perform their routine activities and thus contributes to further economic losses (Mastrandrea *et al.* 2012). The liver was found to be the major organ affected in humans, and this is in agreement with the findings of Fagzel and Ghanbary (2002) and Hong *et al.* (2013)*.*

## Limitations

In this study the prevalence of CE in small ruminants and humans is described. However, the methods employed for detection have several limitations. The reported incidences of human CE are most likely not representative of the true situation as it can be anticipated that most CE patients will not seek medical attention due to the chronic nature of the disease, which is commonly asymptomatic. Similarly, the investigations carried out on small ruminants using routine meat inspection are likely to introduce bias for a number of reasons, such as non-random selection of animals that are sent to slaughter, inability to identify small cysts in their early development stage or limited time available for inspection of each tissue, and uncooperative behaviour of butchers in terms of ensuring thorough meat inspection. In addition, this study does not include the assessment of home slaughter of livestock which (although illegal) is a common practice, especially in rural areas of Ethiopia. Furthermore, no study has been conducted in Ethiopia to assess the sensitivity of the ultrasonographic and abattoir methods for detection of hydatid cysts in humans and animals respectively.

## Conclusion

In conclusion it was found that CE is endemic and relatively prevalent in sheep, goats and humans in central Ethiopia, and also that the high fertility and viability of CE in the liver and lungs suggests that sheep and goats play an important role in the life cycle of this zoonosis. This survey provides preliminary baseline data for further epidemiological studies involving different species of livestock, wildlife and humans in Ethiopia.

Control programmes based on integrated preventive approaches involving improved surveillance of the disease, establishment of well-equipped and standardised abattoirs, use of vaccine in sheep to prevent development of *E. granulosus* cysts, and improving public awareness by means of education, should be implemented for effective prevention of disease transmission. Also, the efficacy of the present methods of diagnosis in animals and humans needs to be evaluated. These initiatives will require additional funding from the government and other agencies to reduce the infection rates in humans and animals. However, the costs and source of funding in developing countries such as Ethiopia constitute a major challenge in implementation of such a CE control programme.
